# CircINTS4 Facilitates Chemoresistance of TNBC by Competitively Binding miR-129-5p/POM121 Axis

**DOI:** 10.1155/2022/2630864

**Published:** 2022-04-04

**Authors:** Qian Tang, Feidu Zhou, Chuanguang Yang, Jue Dai, Jintao Li, Yanxian He

**Affiliations:** Department of Thyroid and Breast Surgery, Liuyang people's Hospital, Hunan, China

## Abstract

**Objectives:**

To detect the expression of circular RNA (circRNA) circINTS4 in triple-negative breast cancer (TNBC) and to analyze the relationship between the expression of circRNA circINTS4 and the clinicopathological characteristics and chemotherapy resistance of patients with TNBC.

**Methods:**

Bioinformatics was used to predict that circINTS4 and POM121 could bind to miR-129-5p, and dual luciferase reporter genes proved that circINTS4 could bind to miR-129-5p and miR-129-5p could bind to POM121. RNA immunoprecipitation (RIP) and RNA pull-down experiments confirmed that circINTS4 binds to miR-129-5p. The correlation among circINTS4, miR-129-5p, and POM121 was detected by qRT-PCR.

**Results:**

In ADR-resistant TNB cells, circINTS4 was significantly up-regulated, miR-129-5p was down-regulated, and POM121 protein expression was significantly up-regulated. Experimental results showed that circINTS4 knockdown inhibited proliferation, migration, invasion, and autophagy. Knocking down miR-129-5p or overexpression of POM121 reversed the inhibitory effect of sh-circints4 on the development of ADR-resistant TNBC cells. In addition, CIRCINTS4 regulates POM121 expression by sponge-adsorbed miR-129-5p. CIRCINTS4 knockdown prevents ADR-resistant tumor growth by regulating the miR-129-5p/POM121 axis in vivo.

**Conclusions:**

CircRNA circINTS4 may act as the ceRNA of miR-129-5p to regulate the expression of target gene POM121, thereby promoting the progress of TNBC molecular mechanism and providing scientific basis for circINTS4 as a new molecular target for clinical diagnosis and drug resistance therapy of TNBC.

## 1. Introduction

In clinical treatment programs, chemotherapy, endocrine therapy, and targeted therapy play an extremely important role as adjuvant treatments for breast cancer patients. However, a large number of clinical practices have shown that these treatment methods encounter the problems of primary and secondary drug resistance, which has become a major treatment obstacle that is difficult to avoid in clinical practice [1, 2]. For example, luminal A and luminal B breast cancers account for 70% to 80% of all types of breast cancer, respectively; approximately 70% of patients had effective initial drug treatment; and approximately 30% of patients had resistance to primary endocrine therapy [3]. Nearly 50% of HER2-amplified breast cancers are resistant to herceptin at the beginning, and secondary resistance appears approximately one year after treatment [4]. Triple-negative breast cancer (TNBC) is more malignant than other types of breast cancer; the drug treatment effect is not ideal; the primary drug resistance ratio is high; the secondary drug resistance time is short; and the prognosis is poor. Therefore, TNBC has been plagued by clinical treatment for a long time. For cancer types, therapeutic drugs with evident effects and small side effects are always limited [5]. The important reason for the development of drug resistance in tumor cells includes the overexpression of antiapoptotic proteins and the impaired apoptosis signal transduction. In addition, TNBC patients are subject to multiple drug resistance, tumor stem cell-mediated tumor resistance, and tumor microenvironment. Although the targets and mechanisms of clinical adjuvant therapy drugs are different, they achieve the purpose of killing tumor cells by inducing apoptosis [6, 7]. In the past, the main idea of overcoming drug resistance is to design drugs for different apoptosis barrier targets to attempt to restore the apoptotic ability of tumor cells. However, due to the extremely complicated mechanism of apoptosis and the inherent heterogeneity of tumor cells, this problem has never achieved major breakthroughs [8].

Recent studies have shown that circular RNA (circRNA), as a new type of noncoding RNA molecule, plays an important role in regulating gene expression. It is a closed loop with no 5' and 3' ends, and its structure makes it more stable in cells. CircRNA has developmental stage specificity and tissue specificity, rich in content, and conserved among species [9]. Increasing evidence shows that circRNA plays an indispensable role in various tumors, such as circFBLIM1, circABCB, and circHIPK3. However, the role of circRNA in the occurrence of TNBC is rarely reported, and most biological functions and underlying mechanisms of circular RNA have not been discovered. In addition, the up-regulation of oncogenic miRNAs causes the down-regulation of tumor suppressor gene expression. On the contrary, the down-regulation of oncogenic miRNAs causes the expression level of oncogenes to increase [10]. Pandolfi et al. put forward the hypothesis of competitive endogenous RNA: noncoding RNA, pseudogenes, and mRNA can compete with miRNA to mutually regulate their genes, providing a new mechanism for gene regulation [11]. Studies have shown that more than four-fifths of circRNA are spliced by exons, and their sequence is the same as that of homologous linear mRNA. circINTS4 promoted proliferation and induced apoptosis in bladder cancer cell lines by regulating the miR-146b/CARMA3 pathway. However, the role of CircINTS4 in TNBC has not been reported; thus, CircINTS4 was selected for the study. In addition, the molecular mechanism of CircINTS4 in tumorigenesis and development remains unclear. This research intended to investigate the influence of circINTS4 in ADR-resistance of TNBC and expound the mechanism of circINTS4 in ADR-resistant TNBC.

## 2. Methods

### 2.1. Patient Tissue Collection and Information

Thirty pairs of clinical specimens of breast cancer were obtained during the operation by the Department of Breast Surgery of Liuyang People's Hospital. Each pair of specimens included breast cancer tumor tissue and normal tissue adjacent to the tumor. Specimens were diagnosed by two independent pathologists. All patients were female with an age range of 27 to 69 years (median age 49 years). None of them had received anticancer therapy (including radiotherapy, chemotherapy, and endocrine therapy) prior to surgery. Before the specimens were obtained, the patients and their family members had agreed and signed informed consent. This study was approved by the Ethics Committee of Liuyang People's Hospital.

#### 2.1.1. Cell Culture

Breast cancer cell lines and epithelial immortalized cells were cultured in 1640 medium containing 10% fetal bovine serum. All cells were cultured in an incubator at 37°C with 5% CO_2_. When the degree of cell fusion is approximately 50%–60%, the cells are replaced with fresh medium 1 hour in advance and placed into the incubator. Serum-free medium and transfection reagent were prepared into mixture A, and serum-free medium and siRNA/microRNA mimics were prepared into mixture B. Mixtures A and B were thoroughly combined and allowed to stand for 15 minutes at room temperature before being added to the cells in the treatment groups. As for the stable infection of lentivirus, the breast cancer cell lines were cultured with 24-well plates. When the cells grew to 50%–60%, the cells were replaced with liquid 1 hour in advance and placed into incubators. Polybrane was mixed with circINTS4 overexpressed lentivirus/negative controls and added to the cells to be infected. After being cultured in the incubator for 24 hours, the medium was changed and placed under fluorescence microscope to observe the transfection efficiency. The cells were screened using the medium containing purinomycin.

#### 2.1.2. Double Luciferase Reporter Gene Assay

293 T cells and target plasmids were prepared for transfection into 96-well plates, and the cell density reached 50%–70%. A 10 *μ*l DMEM was thoroughly mixed with 0.16 *μ*g circINTS4/muta target plasmid and 5 pmol hsa-miR-129-5 and placed at room temperature (solution A). Then, the 10 *μ*l DMEM was thoroughly mixed with 0.3 *μ*l transfection reagent (solution B) and placed at room temperature for 5 min. Mixtures A and B were thoroughly combined with pipetting gun and allowed to stand for 20 minutes. After 48 hours, the cells were collected for detection. The buffer was diluted with distilled water and added to a 96-well plate of 100 *μ*l per well. The cells were blown and placed in a shaker for 15 min. They were moved to a 1.5-ml centrifuge tube, centrifuged at 12000 rpm at 4°C for 10 min, and absorbed the supernatant. A 100 *μ*l luciferase assay reagent II (Progema) working fluid was added to the 96-well plate. Then, 20 *μ*l cell lysate was added to determine the internal reference value. Subsequently, 100 *μ*l STOP & GLO ® Reagent (Progema) was added, blown, and mixed with pipette gun for two to three times, and the luminescence value was measured. This study was approved by the Ethics Committee of Liuyang People's Hospital.

#### 2.1.3. MTT Cell Proliferation Experiment

The cells of each group in the logarithmic growth phase were seeded on 96-well plates at a density of 1 × 105/ml per well. When incubated for 24, 48, and 72 hours, 200 *μ*l of 10% FBS DMEM was replaced, and 20 *μ*l of tetramethyl group was added. Azoazole blue (MTT) solution (5 mg/ml) was incubated at 37°C for 4 hours. Then, the supernatant was discarded; 50 *μ*l of DMSO was added to each well and then shaken at low speed for 10 min. A microplate reader was used to detect the absorbance OD value at a wavelength of 490 nm, and the result was recorded.

#### 2.1.4. Invasion and Metastasis Experiment

After the cells were routinely transfected for 24 hours, the cells were digested and centrifuged. According to the estimated number of cells, 500–3000 *μ*l of serum-free medium was added to resuspend the cells appropriately. A 10 *μ*l of the resuspended cells was mixed with 10 *μ*l trypan blue, and the cells were counted. A 40 *μ*l Matrigel was placed in the transwell chamber of the preinvasion experiment and then stored in the incubator for 1 hour until it solidified prior to use. The transwell chamber of the pretransfer experiment can be managed without any treatment. A 600 *μ*l of serum-containing medium was added to the 24-well plate, and the transwell chamber was placed. Based on the cell density measured earlier, each processed sample was prepared into 200 *μ*l of a mixture containing 20,000 cells. A 200 *μ*l of cell suspension was added to the transwell chamber. The 24-well plate was placed in the incubator for 24 hours. Then, the 24-well plate was removed, the serum medium was aspirated in the well, and the serum-free medium was discarded in the transwell chamber. The transwell chamber was placed on a 600 *μ*l crystal violet for staining and then stored at room temperature for 15 minutes. The crystal violet in the chamber was blown with PBS, and then, a cotton swab was used to wipe the remaining PBS liquid in the chamber. The chamber was placed under a microscope to observe and take pictures.

#### 2.1.5. RT-qPCR Experiment

Trizol kit method was used to extract total RNA from tissues and cells, and reverse transcription kit was implemented to obtain cDNA. The PCR experiment was performed with a real-time fluorescent quantitative PCR instrument, U6 was used as the internal miRNA control, and mRNA was used GAPDH as the internal control. The reaction system is 20 *μ*l, 2× SYBR Mixture is 10 *μ*l, 10 × cDNA template is 1 *μ*l, each of the upstream and downstream primers is 1 *μ*l, and H_2_O is 8 *μ*l. Each sample has three replicates, and the PCR amplification conditions are as follows: 95°C predenaturation for 3 min, 95°C denaturation for 10 s, 60°C annealing for 30 s, 72°C extension for 2 min, 35 cycles, and 72°C extension for 10 min. The product was verified by agarose gel electrophoresis. The threshold value was manually selected at the lowest point of the parallel rise of each logarithmic amplification curve, the *Ct* value of each reaction tube was obtained (threshold cycle), and the 2-*ΔΔ*Ct method was used to analyze the relative quantitative gene expression. Human CircINTS4 Forward:5′-GAAGATGAGATGTATGGGCTC-3′, human CircINTS4 Reverse:5′-AAGTTCCTTGGCACGCTCAT-3′.

miR-129-5p forward, 5′-GGGGGCTTTTTGCGGTCTGG-3′ and reverse, 5′-AGTGCGTGTCGTGGAGTC-3′; human POM121 forward, 5′-CAGAGCACACCGTTTGCCT-3′, and reverse, 5′-GATCCCGCACCAATGGAAAAT-3′; *β*-actin upstream primer: 5′-AGGGGCCGGACTCGTCATACT-3′, downstream primer: 5′-GGCGGCACCACCATGTACCCT-3′; U6 upstream primer: 5′-CTCGCTTCGGCAGCACA-3′, downstream primer: 5′-AACGCTTCACGAATTTGCGT-3′.

#### 2.1.6. Western Blot

The total protein of the tissue and cells was extracted, and the protein concentration was determined according to the BCA test kit instructions. The extracted protein was added to the loading buffer and boiled at 95°C for 10 minutes. 10% polyacrylamide gel electrophoresis was used to separate the protein, and the electrophoresis voltage was 80 V to 100 V. After PVDF transfer, 5% skim milk was sealed at room temperature for 1 hour, and then, the primary antibody MDR1 (1 : 1000, Abcam), POM121 (1 : 2000, Abcam), and GAPDH (1 : 2000, Thermo) were added and incubated overnight at 4°C. After washing, the solution was placed in horseradish peroxide enzyme-labeled secondary antibody (1 : 5000) and incubated for 1 h at room temperature. The fluorescence scanning imaging system was used to obtain the pictures.

#### 2.1.7. RNA Immunoprecipitation (RIP) Experiment

First, the complete lysis buffer (Millipore's Magna RIP™ kit) was configured according to the instructions, the cells were lysed, and the cell lysate was stored at −80°C for subsequent use (only freeze–thaw once). Then, the antibody and magnetic beads were connected, the antibody-magnetic bead suspension was configured, and the thawed samples were mixed and placed on a shaker to incubate at 4°C overnight. When the immunoprecipitation was completed, the suspension was placed on a magnetic stand and washed six times. Finally, the immunoprecipitation products are collected, and RNA was extracted and purified to detect the abundance of target RNA.

#### 2.1.8. Animal Experiments

An MDA-MB-231 cell line (INTS4) with stable and high expression of INTS4 and the cell line (Vector) infected with the control empty virus were used as tools, and nude mice were used as the animal object; 10^5^ cells per mouse were injected through the tail vein. The neck was removed after eight weeks, and the mice were sacrificed. Tumor tissue was collected, and the tumor weight was obtained after taking photos.

#### 2.1.9. Statistical Analysis

All three independent repeated experimental data in the article are expressed as mean ± SD. All data were statistically analyzed using GraphPad Prism 7.0 and SPSS 20.0. The difference was statistically significant with *p* < 0.05. The data involved in the t test are normally distributed, and the variation between groups is similar. RT-PCR detection of clinical samples used Mann–Whitney U test and Wilcoxon test for significance analysis.

## 3. Results

### 3.1. CircINTS4 Is Up-Regulated in ADR-Resistant TNBC Patients

**Figure 1:**
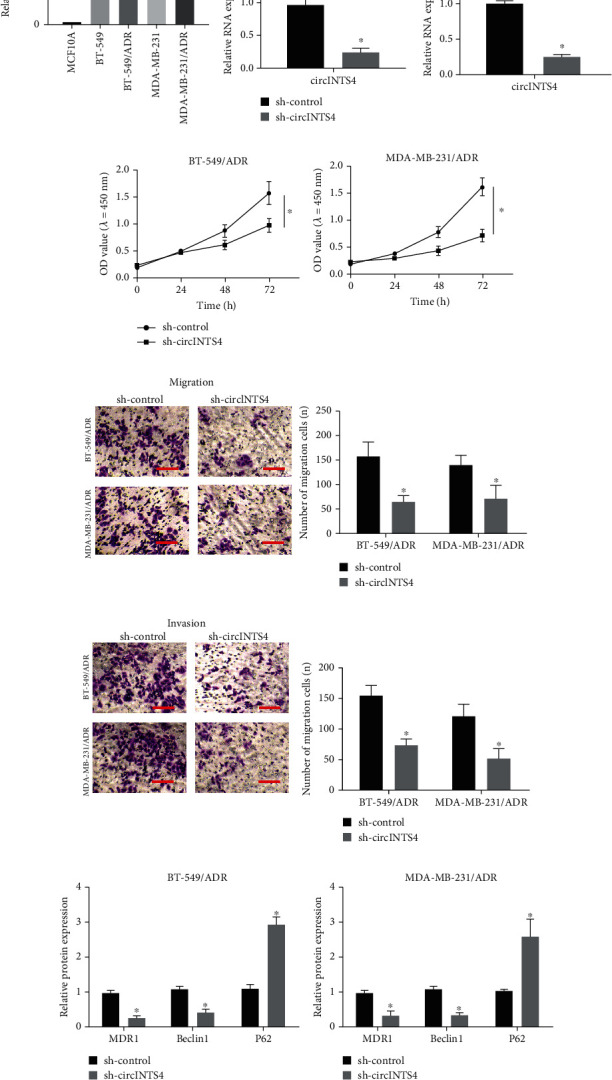
CircINTS4 is up-regulated in ADR-resistant TNBC patients. (a) The expression of CircINTS4 in the parent cell lines and drug-resistant TNBC cell lines. (b) The expression of circINTS4 in BT-549/ADR and MDA-MB-231/ADR cells. (c) MTT was used to test the proliferation ability of BT-549/ADR and MDA-MB-231/ADR cells. (d)–(e). Transwell method was used to test the invasion and migration ability of BT-549/ADR and MDA-MB-231/ADR cells. (f)–(g). The levels of resistance-related proteins MDR1 and autophagy-related proteins Beclin1 and P62 were detected by Western blotting. ∗*p* < 0.05.

We investigated whether circINTS4 is related to TNBC and found that compared with the parent cell lines (MCF10A, BT-549, and MDA-MB-231), the drug-resistant TNBC cell lines (BT-549/ADR and MDA-MB-231/ADR) augmented circINTS4 (Figure 1(a)). The results disclose that circINTS4 imbalance may be related to the resistance of ADR TNBC. In addition, QRT-PCR outcomes unveiled that sh-circINTS4 repressed circINTS4 in BT-549/ADR and MDA-MB-231/ADR cells (Figure 1(b)). Transwell method was implemented to check the migration and invasion potential of drug-resistant TNBC cells. The outcomes unveiled that circINTS4 silencing can diminish the migration and invasion of BT-549/ADR and MDA-MB-231/ADR cells (Figures 1(d) and (e)). In addition, we examined the levels of resistance-related proteins MDR1 and autophagy-related proteins Beclin1 and P62 by Western blot. As unveiled in Figures 1(f) and (g), depletion of circINTS4 can reduce the MDR1 and Beclin1 protein level, and boost the P62 protein level, demonstrating that circINTS4 can also induce autophagy. These results disclose that the depletion of circINTS4 abrogated the progression of drug-resistant TNBC cells.

### 3.2. CircINTS4 Is Directly Related to miR-129-5p

**Figure 2:**
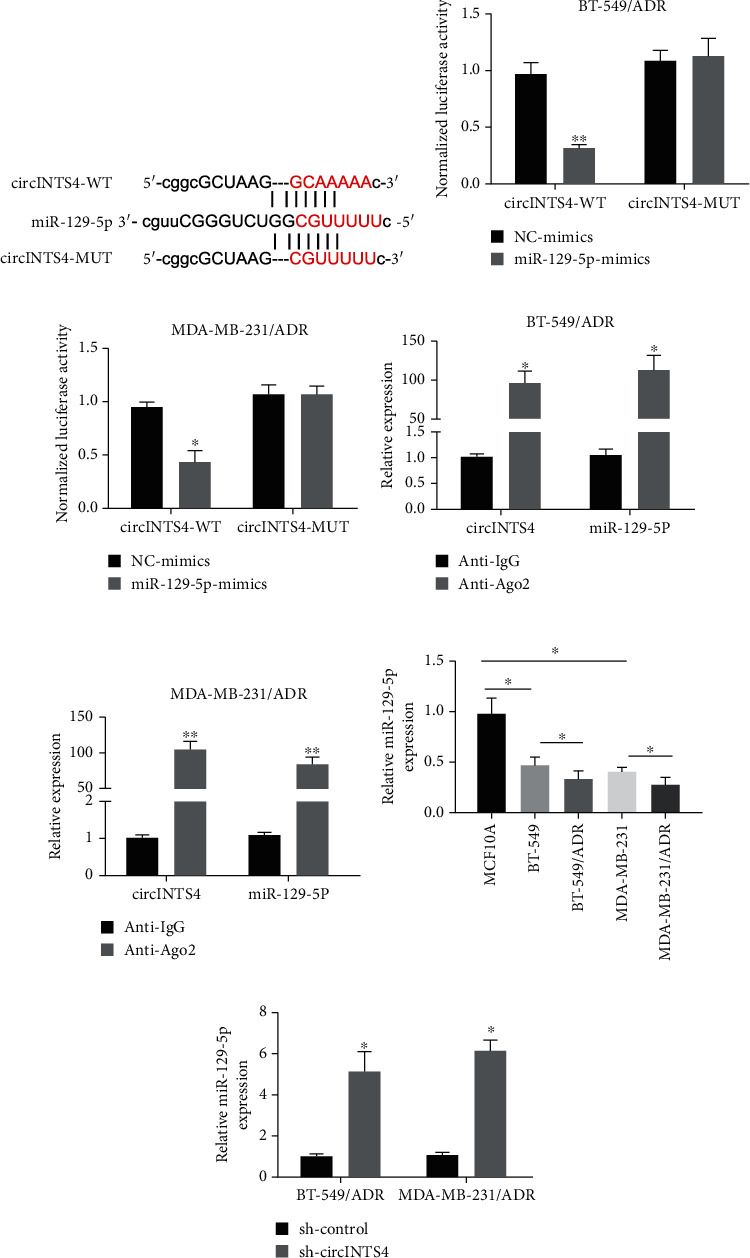
CircINTS4 is directly related to miR-129-5p. (a) Bioinformatics analysis results between CircINTS4 and miR-129-5p. (b) Luciferase reporter gene test was used to test the relationship between CircINTS4 and miR-129-5p in BT-549/ADR cell. (c) Luciferase reporter gene test was used to test the relationship between CircINTS4 and miR-129-5p in MDA-MB-231/ADR cell. (d) RIP experiments. (e) RIP experiments. (f) The expression of miR-129-5p was detected by RT-qCPR. (g) CircINTS4 level was detected by RT-qCPR in BT-549/ADR and MDA-MB-231/ADR cells. ∗*p* < 0.05, ∗∗*p* < 0.01.

Bioinformatic analysis results predicted that circINTS4 was directly related to miR-129-5p (Figure 2(a)). The results of the luciferase reporter gene test unveiled that miR-129-5p-mimics distinctly restrained the luciferase activity of invulnerable cells in the circINTS4-wt group compared with the circINTS4-MUT group (Figures 2(b) and (c)). In addition, RIP experiments unveiled that compared with the anti-IgG group, the levels of circINTS4 and miR-129-5p in the BT-549/ADR and MDA-MB-231/ADR cells of the anti-ago2 group were distinctly accelerated (Figures 2(d) and (e)). Subsequently, we verified that the expression pattern of miR-129-5p in ADR-imperviable TNBC is opposite to circINTS4. We found that miR-129-5p in drug-resistant

TNBC cell lines (BT-549/ADR and MDA-MB-231/ADR) are also repressed relative to the parent cell lines (MCF10A, BT-549, and MDA-MB-231) (Figure 2(f)). In addition, circINTS4 knockdown distinctly accelerated miR-129-5p in BT-549/ADR and MDA-MB-231/ADR (Figure 2(g)). All these data disclose that circINTS4 is directly related to miR-129-5p and negatively regulates its expression in drug-imperviable TNBC cells.

### 3.3. MiR-129-5p Silencing Reverses the Inhibitory Sequel of circINTS4 Knockdown on the Progression of Drug-Resistant TNBC Cells

**Figure 3:**
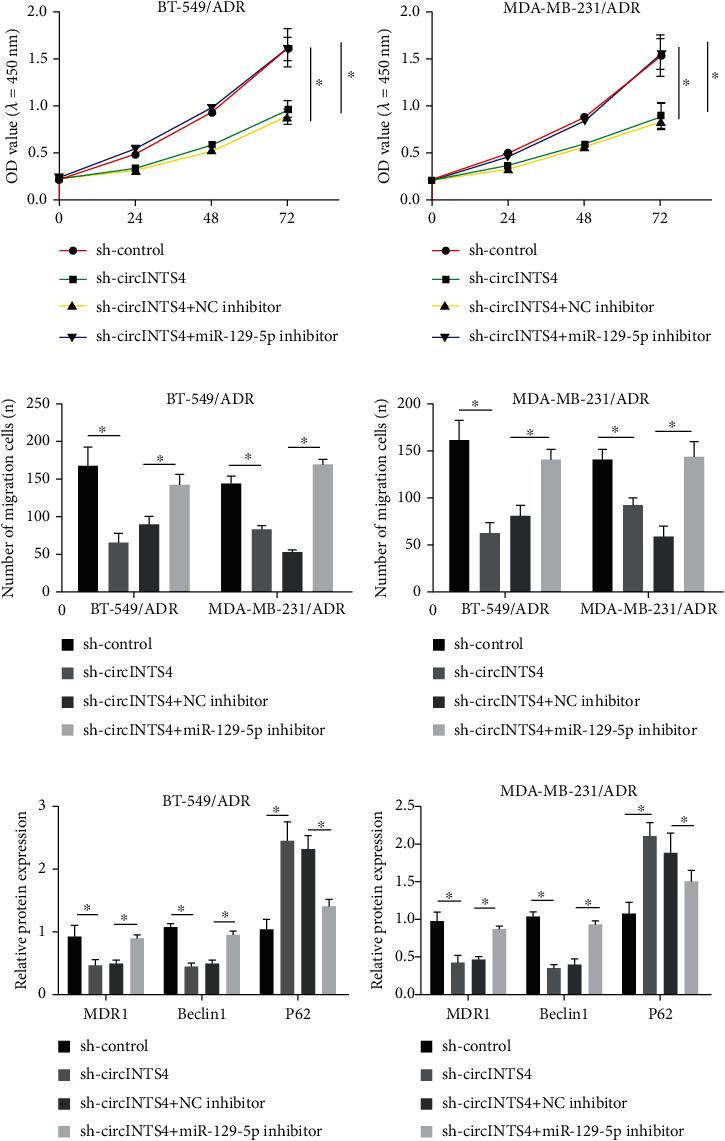
MiR-129-5p silencing reverses the inhibitory sequel of circINTS4 knockdown on the progression of drug-resistant TNBC cells. (a) MTT was used to test the proliferation ability of BT-549/ADR cell. (b) MTT was used to test the proliferation ability of MDA-MB-231/ADR cell. (c) Transwell method was used to test the migration ability of BT-549/ADR cell. (d) Transwell method was used to test the invasion ability of BT-549/ADR and MDA-MB-231/ADR cells. (e) The levels of resistance-related proteins MDR1 and autophagy-related proteins Beclin1 and P62 were detected by Western blotting in BT-549/ADR cell. (f) The levels of resistance-related proteins MDR1 and autophagy-related proteins Beclin1 and P62 were detected by Western blotting in MDA-MB-231/ADR cell. ∗*p* < 0.05.

MTT unveiled that sh-circINTS4 abrogated cell viability, whereas in BT-549/ADR and MDA-MB-231/ADR cells, miR-129-5p inhibitor restrained this sequel (Figures 3(a) and (b)). Transwell experiments unveil that the knockdown of circINTS4 can diminish the migration and invasion of BT-549/ADR and MDA-MB-231/ADR cells, and then, miR-129-5p inhibitors can reverse this process (Figures 3(C) and (D)). In addition, by interfering with miR-129-5p, the sequel of sh-circINTS4 on the decrease in MDRI protein expression, the boost in P62 protein expression, and the decrease in Beclin1 were overturned (Figures 3(e) and (f)). These outcomes disclose that circINTS4 promotes the progression of drug-imperviable TNBC cells by targeting miR-129-5p.

### 3.4. CircINTS4 Positively Regulates POM121 through Sponge-Mediated miR-129-5p

**Figure 4:**
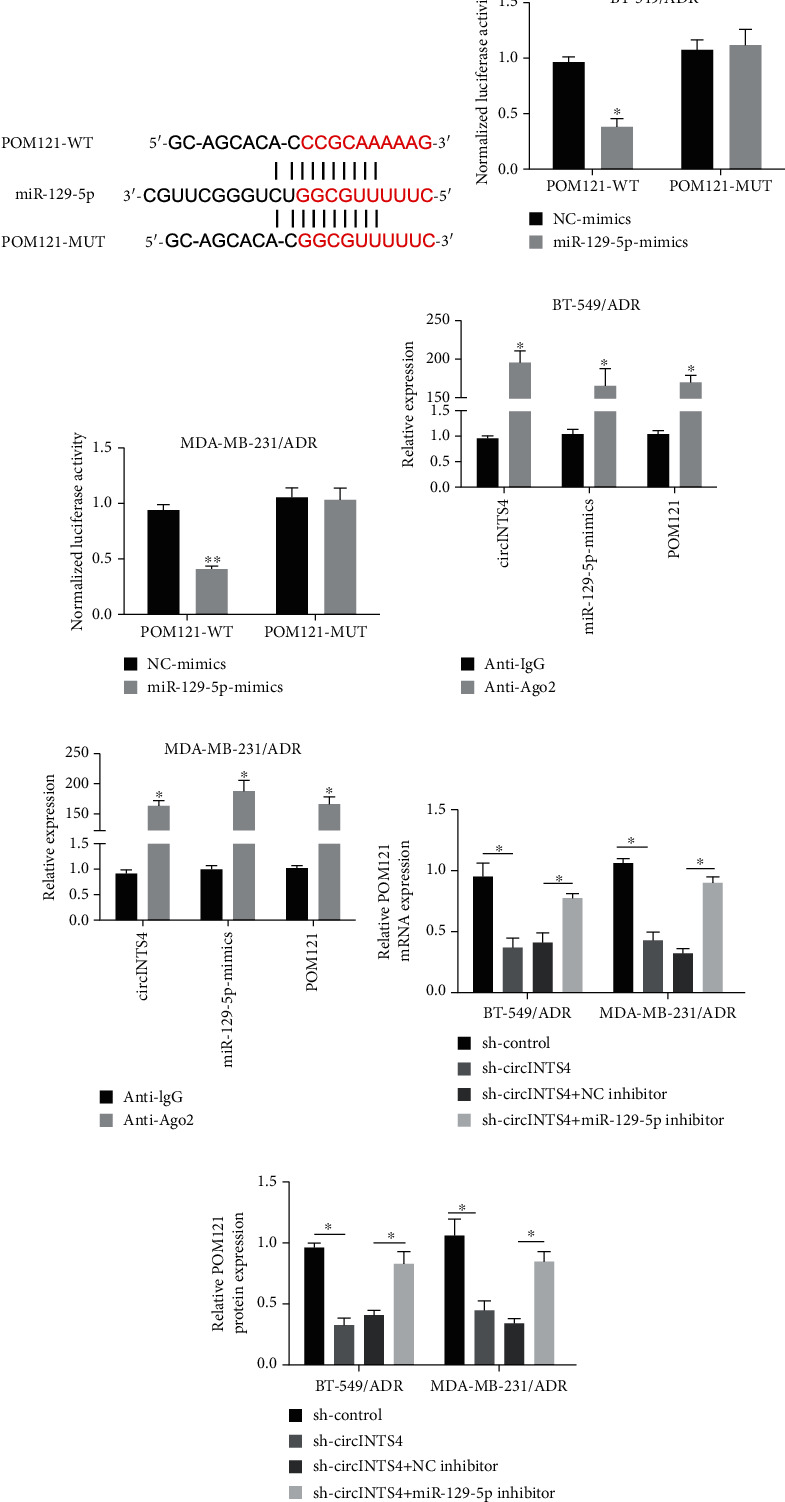
CircINTS4 positively regulates POM121 through sponge-mediated miR-129-5p. (a) Bioinformatics analysis results between POM121 and miR-129-5p. (b) Luciferase reporter gene test was used to test the relationship between POM121 and miR-129-5p in BT-549/ADR cell. (c) Luciferase reporter gene test was used to test the relationship between POM121 and miR-129-5p in BT-549/ADR cell. (d) RIP experiments. (e) RIP experiments. (f) The expression of POM121 was detected by RT-qCPR. (g) POM121 level was detected by RT-qCPR in BT-549/ADR and MDA-MB-231/ADR cells. ∗*p* < 0.05, ∗∗*p* < 0.01.

Then, we found a putative binding site between miR-129-5p and the 3′UTR of POM121 (Figure 4(a)). The luciferase reporter gene assay confirmed the molecular binding between miR-129-5p and POM121 (Figures 4(b) and (c)). Importantly, the outcomes of RIP analysis unveiled that the levels of circINTS4, miR-129-5p, and POM121 in the anti-Ago2 group were distinctly higher than those in the control group (Figures 4(d) and (e)). In addition, circINTS4 silencing can down-regulate the mRNA and protein levels of POM121 in BT-549/ADR and MDA-MB-231/ADR cells, and miR-129-5p inhibitors can reverse these sequels (Figures 4(f) and (g)). In summary, the results exhibit that circINTS4 positively regulates POM121 by sponging miR-129-5p, thereby suggesting the possibility of circINTS4/miR-129-5p/POM121 pathway in anti-ADR TNBC cells.

### 3.5. POM121 Promotes Progression in Drug-Resistant TNBC Cells

**Figure 5:**
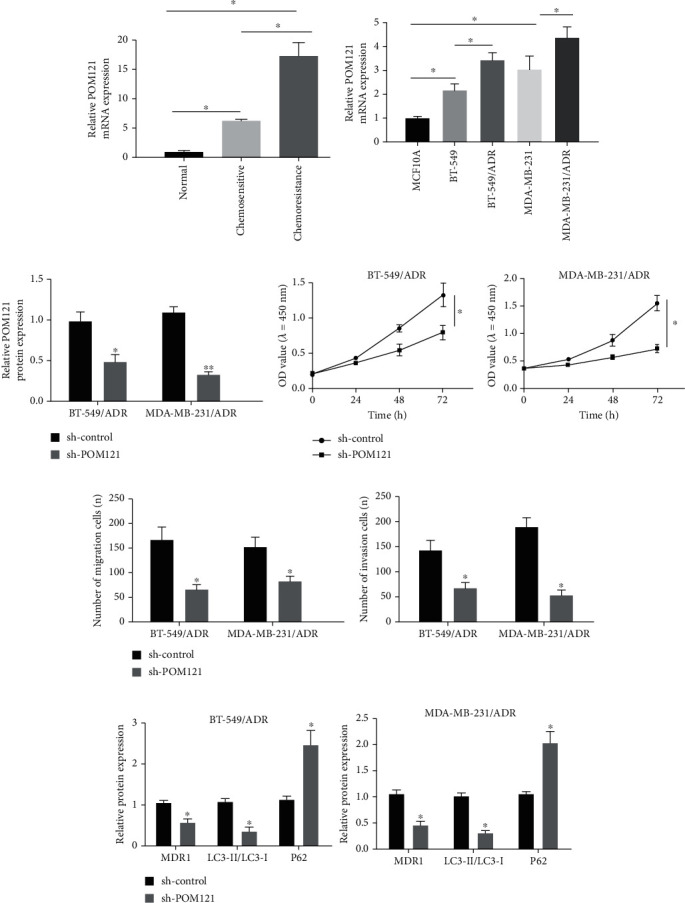
POM121 promotes progression in drug-resistant TNBC cells. (a) POM121 level was tested by RT-qPCR in normal tissues, TNBC tissues, anti-ADR tissues, and ADR chemically sensitive tissues. (b) POM121 level was tested by RT-qPCR in parent cell line and ADR-imperviable TNBC cell lines. (c) POM121 level was tested by RT-qPCR in BT-549/ADR and MDA-MB-231/ADR cells. (d) MTT was used to test the proliferation ability of BT-549/ADR cell. (e) Transwell method was used to test the migration and invasion ability of BT-549/ADR cell and MDA-MB-231/ADR cells. (f) The levels of resistance-related proteins MDR1 and autophagy-related proteins Beclin1 and P62 were detected by Western blotting in BT-549/ADR cell and MDA-MB-231/ADR cell. ∗*p* < 0.05, ∗∗*p* < 0.01.

**Figure 6:**
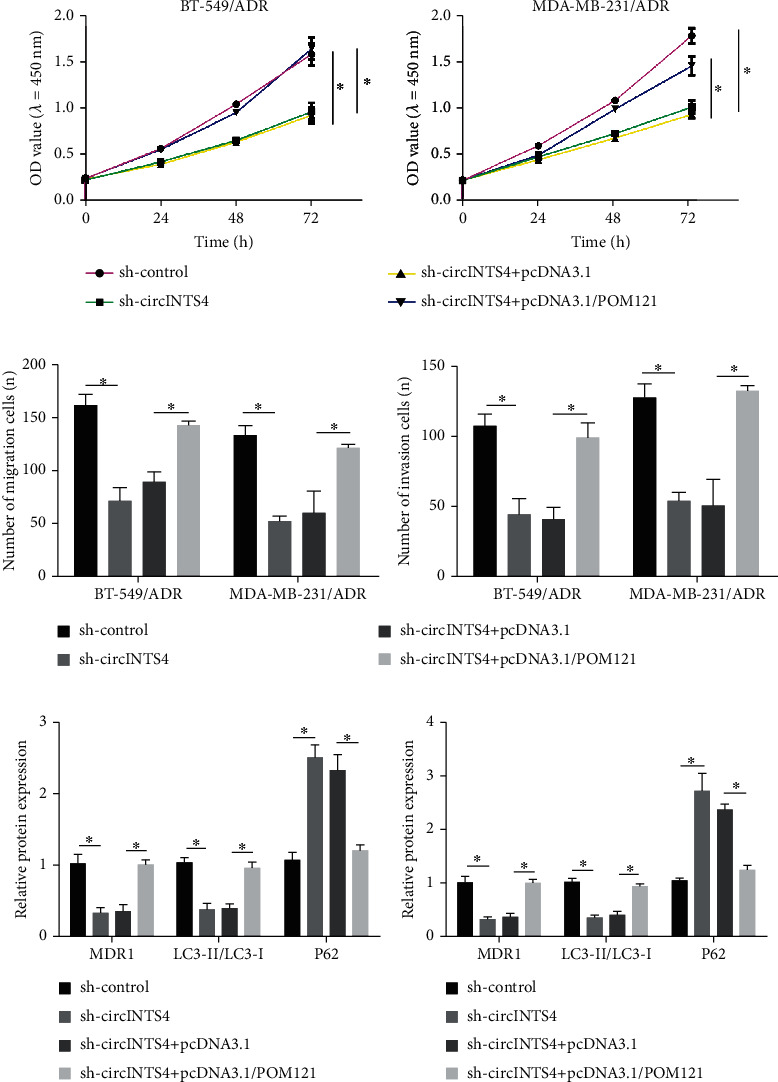
CircINTS4 promotes the development of drug-resistant TNBC cells by up-regulating POM121. (a) MTT was used to test the proliferation ability of BT-549/ADR cell and BT-549/ADR cell. (b) Transwell method was used to test the migration and invasion ability of BT-549/ADR cell and MDA-MB-231/ADR cells. (c) The levels of resistance-related proteins MDR1 and autophagy-related proteins Beclin1 and P62 were detected by Western blotting in BT-549/ADR cell. (d) The levels of resistance-related proteins MDR1 and autophagy-related proteins Beclin1 and P62 were detected by Western blotting in MDA-MB-231/ADR cell. ∗*p* < 0.05.

To study the functional sequel of POM121 on the TNBC process, we examined its expression in TNBC tissues, anti-ADR tissues, and cells. The outcomes of qRT-PCR unveiled that the mRNA levels of POM121 in TNBC tissue and ADR chemically resistant tissue were higher than the normal tissues and ADR chemically sensitive tissues, respectively (Figure 5(a)). Consistently, compared with ADR-imperviable TNBC cell lines (BT-549/ADR and MDA-MB-231/ADR), POM121 has higher protein levels than its parent cell line (MCF10A, BT-549, and MDA-MB-231) (Figure 5(b)). As unveiled in Figure 6(e), relative to the sh-control transfected cells, POM121 protein expression is distinctly restrained in sh-POM121-transfected BT-549/ADR and MDA-MB-231/ADR cells (Figure 5(c)). The results of MTT and transwell analysis exhibited that the interference of POM121 would prevent proliferation (Figure 5(d)), migration, and invasion (Figure 5(e)). In addition, the depletion of POM121 restrained the level of MDR1 protein, augmented the level of P62 protein, and restrained Beclin1 (Figure 5(f)). These data indicate that the depletion of POM121 attenuated the progression of TNBC.

### 3.6. CircINTS4 Promotes the Development of Drug-Resistant TNBC Cells by Up-Regulating POM121

Subsequently, we further studied the interaction of circINTS4 and POM121 on the progression of TNBC. Western blot was implemented to check POM121 in cells transfected with POM121. The MTT results exhibited that overexpression of POM121 attenuated the inhibitory sequel of sh-circINTS4 on the proliferation of BT-549/ADR and MDA-MB-231/ADR cells (Figures 6(a) and (b)). The outcomes of transwell experiments unveil that circINTS4 silencing can attenuate migration and invasion of BT-549/ADR and MB-231/ADR cells by up-regulating POM121 (Figure 6(b)). Concurrently, when sh-circINTS4 was transfected into BT-549/ADR and MDA-MB-231/ADR cells, P62 was accelerated, MDR1 was dwindled, and Beclin1 decreased. However, the overexpression of POM121 overturned the pair of sh-circINTS4 and the impact of these protein levels (Figures 6(c) and (d)). These results disclose that circINTS4 knockdown attenuated TNBC progression by regulating POM121.

### 3.7. CircINTS4 Promotes Tumor Growth of Drug-Resistant TNBC Cells by Regulating the miR-129-5p/POM121 Axis In Vivo

**Figure 7:**
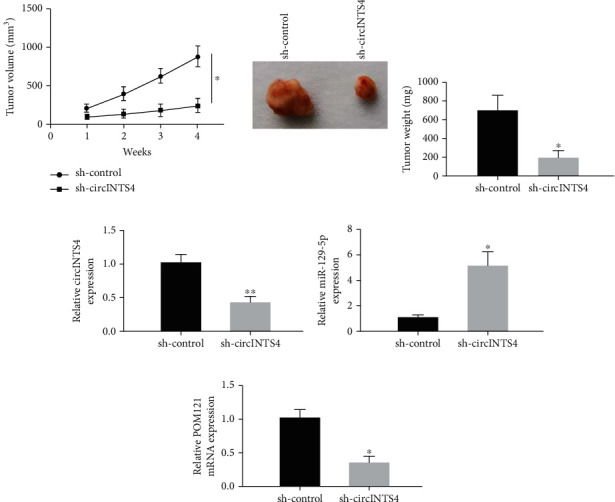
CircINTS4 promotes tumor growth of drug-resistant TNBC cells by regulating the miR-129-5p/POM121 axis in vivo. (a) Tumor volume diagram. (b) Tumor weight histogram. (c)–(d). RT-qCR was used to detect the expression of CircINTS4, miR-129-5p, and POM121. ∗*p* < 0.05, ∗∗*p* < 0.01.

Then, MDA-MB-231/ADR cells in vivo were used to perform xenotransplantation mice experiments to check the sequel of circINTS4 on tumor growth. The results exhibited that circINTS4 knock down distinctly restrained tumor volume (Figures 7(a) and (b)). RT-qCR results unveiled that sh-circINTS4 repressed the level of circINTS4 in tumor tissues resected in nude mice (Figure 7(c)). Subsequently, we found that miR-129-5p was augmented in vivo (Figure 7(d)), whereas at the mRNA levels, knockdown of circINTS4 resulted in depletion of POM121 (Figure 7(e)). These data indicate that circINTS4 silencing attenuates tumor growth of drug-imperviable TNBC cells by regulating the miR-129-5p/POM121 axis in vivo.

## 4. Discussion

TNBC accounts for 10%–20% of breast cancers and is more common in young women. It is characterized by loss of expression of estrogen receptor (ER), progesterone receptor (PR), and human epidermal growth factor receptor 2 (HER2) [12]. Given its characteristics, TNBC patients can hardly benefit from molecular targeted therapy or endocrine therapy. Compared with other types of breast cancer, TNBC usually appears larger, more malignant, and more aggressive [13]. Some TNBCs have original resistance at the beginning of treatment, even if they may be sensitive to chemotherapy in the early stage. Studies in recent years have shown that in addition to apoptosis, tumor cells can die through backup death mechanisms, such as programmed necrosis [14], and programmed necrosis cannot be avoided by tumor cells.

After discovering that miRNA is related to cancer, the same research enthusiasm is observed on circRNA [15, 16]. In a short period of time, many circRNAs were found to be associated with various solid and blood malignancies. The research results of repressed/up-regulated circRNAs in cancer can be found in some articles [17–19]. From the effects of circRNAs reported in at least two journals on the same/different types of tumors, only a very small amount of circRNAs satisfies these conditions. Most reported cancer-related circRNAs are related to gastrointestinal malignancies. Some researchers have found that CDR1as is up-regulated in hepatocellular carcinoma (HCC) compared with normal adjacent tissues, and miR-7 is negatively correlated with CDR1as. In vitro experiments have shown that CDR1as is an oncogene that can increase the proliferation and invasion potential of cancer cells by adsorbing miR-7 [20]. Recently, another set of experiments analyzed the levels of CDR1as in HCC tissues and paired adjacent normal tissues, and circular RNAs were observed to have lower expression in malignant tissues. In addition, some circRNAs were related to tumors. Studies have shown that through in vivo and in vitro experiments, circ-Foxo3 is a potential tumor suppressor in breast cancer; it works by using miRNA sponge [21]. HIPK3 is a tumor suppressor with protein kinase activity. Studies have shown that in HCC, circHIPK3 is increased, and it can act as a sponge for nine different miRNAs. Knockdown of circHIPK3 leads to decreased proliferation ability [11]. CircRNA_001569 is a carcinogen in CRC; the researchers found that circ_001569 is up-regulated in tumors, is related to the TNM staging of tumors, and works by adsorbing miR-145 [22].

We used bioinformatics methods for prediction, and through qRT-PCR methods, we found that circINTS4 can negatively regulate miR-129-5p. Through Western blotting, circINTS4 can still regulate the expression level of POM121 protein. Through dual luciferase detection, RNA pull-down test, and RNA immunoprecipitation (RNA Immunoprecipitation, RIP) test, we confirmed that circINTS4 contains the same target as miR-129-5p and can be powerful with miR-129-5p. We also found that circINTS4 in breast cancer cells was significantly higher than that in epithelial immortalized cells. In the study of cell biological function, in vitro experiments show that down-regulating circINTS4 can significantly inhibit cell proliferation, invasion, and metastasis. In vivo tumor formation experiments in nude mice, overexpression of circINTS4 can significantly promote the growth of subcutaneous tumors. In the study of the experimental mechanism, we found that circINTS4 can act as an endogenous competitive RNA to sponge miR-129-5p, thereby releasing the inhibitory effect of miR-129-5p on POM121 expression.

POM121 is a member of the 30 nuclear pore complex protein family. It is a membrane-anchored nuclear pore complex protein that anchors the nuclear pore complex on the nuclear membrane by interacting with other nuclear pore complex subunits [23]. The N-terminus of POM121 has a potential transmembrane domain, and the C-terminus contains a domain characteristic of the nuclear pore complex, such as the complex and disordered FG repeat sequence [24]. Immunofluorescence of rat liver cells showed the dot-like distribution of POM121 on the nuclear membrane, and immunoelectron microscopy techniques revealed the location of POM121 and other nuclear pore complex proteins [25]. Knockdown of POM121 by siRNA can lead to the clustering of nuclear pore complexes in Hela cells. A literature has reported that the fusion expression of the two proteins caused by the ectopic expression of PAX5 and POM121 genes leads to acute lymphoblastic leukemia [26], and POM121 is involved in the assembly process of nuclear pores during the nuclear membrane formation during the division cycle.

miRNA is a class of noncoding gene sequences with a conserved structure widely present in eukaryotic organisms. More general miRNAs are located on chromosomal fragile sites and participated in the process of cell canceration. The miR-129 family includes two member molecules, miR-129-3p and miR-129-5p. Studies have confirmed that miR-129 family molecules can promote the occurrence or development of many malignant tumors and belong to tumor suppressor miRNAs. Zhang Hui et al. [27] proved that miR-129-5p inhibits the proliferation of osteosarcoma cells and promotes apoptosis by inhibiting p-GSK-3*β* and *β*-catenin. He Bingsheng et al. [28] found that miR-129 can affect the self-renewal ability of breast cancer stem cells by regulating the Notch signaling pathway in breast cancer. The specific regulatory mechanism may be the down-regulation of Let-7b expression caused by ESR1, which leads to Numb. The release eventually inhibits the activation of the Notch signaling pathway. A new target gene candidate of miR-129-5p, POM121 was searched through the biological website TargetScan, and its effect on the proliferation and invasion of breast cancer cells was evaluated. The QPCR results showed that miR-129-5p in breast cancer cell lines MDA-MB-231/ADR and BT549/ADR was significantly lower than that in normal breast epithelial cells MCF-10A. The change in miR-129-5p is related to the canceration of breast epithelial cells. After up-regulating miR-129-5p, the proliferation, migration, and invasion activities of MDA-MB-231/ADR cells were significantly reduced, thereby confirming that miR-129-5p played a similar activity to tumor suppressor genes in the malignant biological behavior of breast cancer cells.

## Data Availability

The analyzed data sets generated during the study are available from the corresponding author on reasonable request.
